# Epigenomics of AD: a focus on DNA methylation and microRNAs

**DOI:** 10.1002/alz.083399

**Published:** 2025-01-03

**Authors:** Katie Lunnon

**Affiliations:** ^1^ University of Exeter, Exeter, Devon United Kingdom

## Abstract

Alzheimer’s disease (AD) is a multi‐factorial and complex disease, with the risk of developing disease still largely unknown despite numerous genetic and epidemiological studies over recent years. Several genetic and modifiable lifestyle risk factors are known to contribute to disease etiology, and epigenetic mechanisms are suggested to also contribute my mediating their interaction. It is now ten years since we published the first cross‐tissue epigenome‐wide association study (EWAS) of DNA methylation in AD post‐mortem brain samples, with subsequent studies nominating robust and reproducible alterations in genes such as *ANK1*, *HOXA3* and *RHBDF2*. We have leveraged these studies to perform large‐scale epigenomic meta‐analyses, reporting 220 differentially methylated loci that are altered in AD cortex, and which we are currently comparing to signatures in other neurodegenerative diseases. More recently we have undertaken DNA methylomic studies in blood in a quest to identify disease‐associated alterations in an easily accessible tissue, which could be potentially explored from a biomarker perspective. Finally, our most recent work has explored the contribution of non‐coding RNAs to regulating gene expression in AD cortex, where we have meta‐analyzed microRNA expression in ∼1,000 post‐mortem cortical brain samples, establishing cell‐type specific disease‐associated signatures.

